# Editorial

**DOI:** 10.2478/v10102-011-0001-2

**Published:** 2011-03

**Authors:** Michal Dubovický

**Affiliations:** Institute of Experimental Pharmacology & Toxicology, Slovak Academy of Sciences, Bratislava, Slovak Republic. E-MAIL: michal.dubovicky@savba.sk

Due to the intensive impact of chemicals on the environment, human populations and all living organisms have been exposed to various kinds of chemical substances. The number of new chemical compounds produced every year keeps increasing. Many of these so called xenobiotics are highly active substances and may have a significant effect on the living system. After entering the body system, they can affect many biochemical and physiological processes. In the case of new drugs, the effects should be desirable, i.e. protective and/or curative. However, the use of drugs, depending on their dose, may exhibit also undesirable and even toxic effects. It is therefore inevitable to investigate not only the effects of drugs and their fate in the organism (pharmacodynamics and pharmacokinetics) but also to assess their possible unfavorable influences on the living system (toxicology). In the case of industrial chemicals emitted to the environment, as well as chemical substances used in working places, households, it is essential to evaluate their potential toxic effects on humans, animals and plants. Recent pharmacology and toxicology must challenge the increasing demands of the society for safe drugs and other chemicals used by humans. This requires close cooperation of pharmacologists and toxicologists from the academic and industrial sphere as well as professionals from the field of regulatory toxicology and legislation. Up-to-date approaches and techniques have to be utilized in preclinical and safety assessment studies. Due to the tens of thousands of new chemical substances produced, low- and medium-through-put tests will have to be replaced eventually by high-through-put alternative methods using lower vertebrates, non-vertebrates or *in vitro* studies.

The state of the art and development of pharmacology and toxicology in the Slovak Academy of Sciences and Universities in Slovakia is presented in this issue of Interdisciplinary Toxicology. Individual articles are proceedings of the lectures given at the symposium “Recent Slovak Pharmacology and Toxicology” held on April 5, 2011, at the campus of the Slovak Academy of Sciences in Bratislava. The papers are focused on pharmacological modulation of the activity of phagocytes and neutrophils, immunobiological properties of modified polyphenols and isoprenoids, on the regulation of immunity and oxidative stress in the model of rheumatoid arthritis, they further concern studies of effects and safety of original pyridoindoles, experimental model of metabolic syndrome, the pharmacologic modulation of allergic asthma, as well as vascular pharmacology and protection of the endothelium in experimental conditions. Many results presented have been achieved in close cooperation with Czech scientists.

The symposium and the issue of the Journal are dedicated to the birthday anniversary of the outstanding Slovak pharmacologist, Professor Radomír Nosál, MD., DSc., a founder and key personality in cell pharmacology in Slovakia, as well in the former Czecho-Slovakia. He is a great propagator and supporter of toxicology in Slovakia.

The Editorial Board wishes to Professor Radomír Nosal many happy returns of the day and much further success.

